# Improving Pediatric Academic Global Health Collaborative Research and Agenda Setting: A Mixed-Methods Study

**DOI:** 10.4269/ajtmh.19-0555

**Published:** 2020-01-13

**Authors:** Chris A. Rees, Elizabeth M. Keating, Kirk A. Dearden, Heather Haq, Jeff A. Robison, Peter N. Kazembe, Florence T. Bourgeois, Michelle Niescierenko

**Affiliations:** 1 Division of Emergency Medicine, Boston Children’s Hospital, Harvard Medical School, Boston, Massachusetts;; 2 Division of Pediatric Emergency Medicine, University of Utah, Salt Lake City, Utah;; 3 IMA World Health, Dar es Salaam, Tanzania;; 4 Department of Pediatrics, Baylor College of Medicine, Houston, Texas;; 5 Baylor College of Medicine Children’s Foundation Malawi, Lilongwe, Malawi

## Abstract

Academic global health collaborations have the potential to improve joint understanding of health issues in low- and middle-income countries (LMICs). Our objective was to elucidate perceptions of benefits and challenges of academic global health collaborations as well as areas for improving collaborative research conducted in LMICs. This cross-sectional, mixed-methods study surveyed investigators’ perceptions of benefits and challenges of pediatric academic global health collaborations. Authors of articles from four pediatric journals reporting pediatric research conducted in LMICs published between 2006 and 2015 were surveyed. Responses of LMIC investigators were compared with those of investigators in high-income countries (HICs). Responses to open-ended questions were analyzed using a combined thematic and content analysis approach. Of 1,420 potential respondents, 252 (17.7%) responded to the survey. Collaborative research with investigators from other countries was perceived as beneficial by 88.5% of respondents (*n* = 223), although this perception was more common among HIC respondents (*n* = 110, 94.0%) than LMIC respondents (*n* = 113, 83.7%) (*p* = 0.014). Sixty-seven percent (*n* = 170) of respondents perceived that HIC investigators had set the research agenda in work conducted in a LMIC. Respondents identified several critical factors to improve academic global health collaborations, including research capacity building, communication, and early involvement of LMIC investigators with shared decision-making during study conception and grant writing. Pediatric academic global health collaboration was widely perceived as positive. However, despite calls for capacity building and locally generated research ideas, many respondents felt that HIC investigators set the research agenda for work conducted in LMICs. This study provides suggestions for improving collaboration among pediatric academicians globally.

## INTRODUCTION

Research conducted in low- and middle-income countries (LMICs) often involves collaboration between investigators from high-income countries (HICs) and investigators from LMICs.^1^ Academic global health collaborations have increased in recent years and have the potential to improve joint understanding of health issues in LMICs.^2–4^ Academicians who engage in global health research often believe that collaborations between HICs and LMICs are central to research conducted in LMICs.^5^ Benefits from such collaboration include LMIC investigator access to grants from HICs, the diffusion of evidence-based health policy to LMICs, improved cross-cultural communication between HIC and LMIC collaborators, and innovations developed in LMICs that benefit patients and providers in both LMICs and HICs.^6–9^

Despite these benefits, the relationships are also beset with challenges. Global health research has been called a “field of power relations” because of the imbalances in influence between LMIC and HIC investigators.^10^ Some academicians have argued that the popular shift in terminology from collaboration to “partnership”^11–13^ may simply reflect “postcolonial anxieties” among HIC collaborators.^14^ Partnerships between LMIC and HIC investigators are sometimes paternalistic, and academic global health collaborations may unequally benefit HIC investigators when compared with LMIC investigators.^15,16^

Case studies and opinion pieces describing challenges and successes in single partnerships between LMIC and HIC investigators have been published previously,^8,17–20^ and policies have been put forth to establish guidelines for equitable academic global health collaborations.^21–23^ However, previously published studies on perceptions of academic global health collaborations have been limited to single partnerships, and policies for equitable academic global health collaborations have been based on a paucity of evidence.^24^ To support effective and mutually beneficial academic global health collaborations, a deeper understanding of the experiences and perceptions of researchers engaged in these collaborations is needed. Our objective was to elucidate pediatric investigators’ perceptions of benefits and challenges of academic global health collaborative work conducted in LMICs. In addition, we sought to identify potential areas of improvement in such collaborations.

## MATERIALS AND METHODS

### Study design.

We conducted a cross-sectional, mixed-methods, online survey. This study was exempted from ethical review by the Boston Children’s Hospital Institutional Review Board.

### Participants.

We surveyed corresponding and non-corresponding authors of original research reporting work conducted in LMICs that was published in the four leading pediatrics journals according to the Eigenfactor score in 2016 (*Pediatrics*, *Journal of Pediatrics*, *Pediatric Infectious Disease Journal*, and *Pediatric Blood and Cancer*).^25^ We reviewed all articles reporting original research conducted in LMICs (as defined by the World Bank)^26^ and published in these four journals from January 1, 2006 to December 31, 2015.

Authors’ affiliations (including affiliated country) at the time of article publication, email addresses, reported degrees, and authorship position in the reviewed articles were extracted. We used an online random number generator to select a single, non-corresponding author from each article to create a more representative sample of both LMIC and HIC investigators.^27^ Once the non-corresponding author from each article was selected, we searched for the authors’ name in PubMed, Google Scholar, and Google to obtain non-corresponding authors’ email addresses when possible. If an email address for the non-corresponding authors was not identified through this approach, the author was determined to be ineligible for the study.

### Survey development.

We designed a survey instrument to collect data on respondents’ demographics and their impression of benefits and challenges of academic global health collaborations (Supplemental Appendix 1). Survey questions were developed using an iterative process among this study’s authors until consensus was achieved. The survey was also reviewed by a group with survey-development expertise to ensure readability of survey questions and to minimize bias in question wording. Survey questions were written in English as all authors had published in English-only journals.

### Survey distribution.

The survey was sent to potential respondents via email through REDCap, a secure web application.^28^ As per the standard methodology for online surveys,^29,30^ two reminder emails were sent weekly to potential participants who did not complete the survey. Consent was implied through respondent completion of the survey. Three $100 Amazon gift cards were offered as incentives to respondents. We excluded surveys with partial responses (*n* = 3). Questions for this study followed a survey assessing perceptions of authorship in academic global health publications.^31^ At the beginning of this survey, respondents confirmed they had collaborated with investigators in countries other than their own to qualify for the survey.

### Analysis.

We used descriptive statistics to describe respondents’ demographic characteristics. Respondents were categorized as LMIC respondents or HIC respondents based on their self-reported country of residence at the time of the survey. As the aim of our study was to describe perceptions of pediatric academic global health collaboration, we assumed respondents’ current environment, denoted by their current countries, would more accurately reflect respondents’ recent experience and insights than would their birth country. To assess the frequency of respondents who may have been born in one setting but later migrated to another, we calculated the proportion of respondents who had discrepant reported birth and countries of residence at the time of the survey. To assess for sampling bias, using available aforementioned data, we compared respondents’ and nonrespondents’ country of affiliation, mean authorship position, and corresponding author status. All comparisons between LMIC and HIC responses were made using the Pearson chi-squared statistic. Based on the response rate from a similar online survey study,^32^ we anticipated a response rate between 15% and 20%. As such, we sent the survey to 1,420 authors, anticipating a minimum of 200 responses. At the beta level of 0.8 and alpha of 0.05, our sample was powered to detect at least a 10% difference in responses from LMIC respondents and HIC respondents using the Pearson chi-squared statistic.

Responses to open-ended questions regarding benefits, harms, and ways to improve academic global health collaborations were analyzed thematically. Responses to open-ended questions soliciting opinions regarding steps to improve collaborative research efforts between LMIC and HIC investigators were analyzed using a combined thematic and content analysis approach by counting the frequency of themes found in respondents’ quotes. Atlas.ti (version 8.1.3, ATLAS.ti Scientific Software Development GmBH, Berlin, Germany) was used for thematic and content analysis. Two authors (C. A. R. and H. H.) used inductive thematic analysis to independently code the open-ended responses. The two authors then reconciled themes that emerged and, using constant comparison, developed themes from the open-ended responses.^33^

## RESULTS

The survey was sent to 1,420 potential respondents, and 252 (17.7%) responded. Of the 252 respondents, 53.6% (*n* = 135) were living in LMICs and 46.4% (*n* = 117) were living in HICs at the time of survey completion (Table 1). Of the 135 respondents living in LMICs, 70.4% (*n* = 95) were living in upper middle–income countries, 22.2% (*n* = 30) in lower middle–income countries, and only 7.4% (*n* = 10) in low-income countries. Seventy-five percent (75.4%, *n* = 190) of respondents reported the same current and birth countries, and 24.6% (*n* = 62) had different current countries than their birth countries. Of the 62 respondents who reported one birth country and another current country, 48.3% (*n* = 30) were in countries with the same World Bank income group designation as their birth country. Only 12.7% (*n* = 32) of all respondents reported having been born in countries from a different World Bank group than that of their current location.

**Table 1:** Demographic characteristics, training, and employment of respondents

Characteristic	All respondents (*n* = 252)		Respondents living in HICs (*n* = 117)		Respondents living in LMICs (*n* = 135)		
	*n*	%	*n*	%	*n*	%	*P*-value*
Female	127/252	50.4	62/117	53.0	65/135	48.1	0.443
Age (years), median	51.5	IQR (43.75–60)	53	IQR (44–61)	49	IQR (43–59)	
World Bank region for country of birth							< 0.001
HIC	127/252	50.4	107/117	91.5	20/135	14.8	
Upper middle–income country	87/252	34.5	4/117	3.4	83/135	61.5	
LMIC	28/252	11.1	4/117	3.4	24/135	17.8	
Low-income country	10/252	4.0	2/117	1.7	8/135	5.9	
English is a primary language	120/252	47.6	78/117	66.6	42/135	31.1	< 0.001
Highest degree							0.005
Medical doctorate (MD, MBChB, and MBBS)	90/252	35.7	53/117	45.3	37/135	27.4	
Doctorate degree (Doctorate; MD and doctorate; and MBChB, MBBS and doctorate)	152/252	60.3	62/117	53.0	90/135	66.7	
Masters degree and other†	10/252	4.0	2/117	1.7	8/135	5.9	
Medical training or nonmedical highest degree completed in							< 0.001
HIC	139/252	55.2	104/117	88.9	35/135	25.9	
LMIC	113/252	44.8	13/117	11.1	100/135	74.1	
Current position							0.015
Professor, clinical instructor, and lecturer	214/264	81.1	96/126	76.2	118/138	85.5	
Medical doctor with no academic appointment, medical officer	14/264	5.3	5/126	4.0	9/138	6.5	
Employed by nongovernmental organization, resident, fellow, and other‡	36/264	13.6	25/126	19.8	11/138	8.0	
Number of peer-reviewed articles (median)	65	IQR (30–120)	80	IQR (33–150)	53	IQR (25–106)	

HICs = high-income countries; LMICs = low- and middle-income countries.

* Comparison is between authors living in HICs and those in LMICs. Test statistic is Pearson χ^2^ test.

† Other highest degrees included PharmD (*n* = 1) and Thai Board of Radiology (*n* = 1).

‡ Other current positions include student (*n* = 4), reader (*n* = 2), clinical officer (*n* = 2), employed by pharmaceutical company (*n* = 3), support staff (*n* = 2), and other (*n* = 23).

Respondents and non-respondents did not differ by whether they were the corresponding author in reviewed articles (*p* = 0.631), mean authorship position in reviewed articles (respondents’ mean authorship position = 3 ± 3.3 versus non-respondents’ mean authorship position = 3.8 ± 3.5, *p* = 0.167), or degrees listed (*p* = 0.884) (Supplemental Table 1). However, respondents did differ from non-respondents by country designation as listed in author affiliation. A greater proportion of respondents had HIC-listed affiliations (*n* = 111, 44.0%) than non-respondents (*n* = 400, 34.2%), and respondents had a lower proportion of upper middle–income affiliations (*n* = 97, 38.5%) and lower middle–income affiliations (*n* = 31, 12.3%) than non-respondents (*n* = 505, 43.2% and *n* = 204, 17.5%, respectively, *p* = 0.018).

Collaborative research with investigators from countries other than the investigator’s own was perceived as beneficial by 88.5% of total respondents (*n* = 223) (Table 2). This perception was more common among respondents living in HICs (*n* = 110, 94.0%) than among those living in LMICs (*n* = 113, 83.7%) (*p* = 0.014). In qualitative responses to an open-ended question about the benefits of collaborative research in LMICs, respondents from both LMICs and HICs recognized bidirectional exchange of ideas and knowledge, increased publications, more opportunities for career advancement, and added ability to address critical health problems in varied settings through long-term personal connections. Both LMIC and HIC respondents commented on the added value of different perspectives and skills among investigators from settings other than their own. One LMIC respondent stated:

The dialogue enriches the methodological design and the discussion of the results. Different views of a problem are core to scientific development.

**Table 2:** Perceptions of international collaboration in pediatric research conducted in LMICs

	All respondents		Respondents living in HICs		Respondents living in LMICs		
	*n*	%	*n*	%	*n*	%	*P*-value*
Collaborating with researchers from other countries perceived as *beneficial*	223/252	88.5	110/117	94.0	113/135	83.7	0.014
Collaborating with researchers from other countries perceived as *harmful*	22/252	8.7	11/117	9.4	11/135	8.1	0.725
Perception that high-income author(s) defined research agenda	170/252	67.5	82/117	70.1	88/135	65.2	0.407
Perceived reason that high-income author(s) defined research agenda							
They had access to funding for the study	138/252	54.8	69/117	59.0	69/135	51.1	0.211
They had the idea for the study	121/252	48.0	72/117	61.5	49/135	36.3	< 0.001
They had published on that topic previously	81/252	32.1	47/117	40.2	34/135	25.2	0.011
They had the time to complete the study	48/252	19.0	29/117	24.8	19/135	14.1	0.032
They had resources at their institution to complete the study	95/252	37.7	53/117	45.3	42/135	31.1	0.021
Other†	7/252	2.8	1/117	0.9	6/135	4.4	0.083

HICs = high-income countries; LMICs = low- and middle-income countries.

* Comparison is between authors living in HICs and those in LMICs. Test statistic is Pearson χ^2^ test.

† Other reasons included “they were more insightful than other team members and able to define the research agenda consistent with national priorities” (*n* = 1), “they had to comply with their organization’s rules” (*n* = 2), “often researchers from HICs use ideas, research questions, and even protocols that arise in low-income countries which then use them to promote their own research agenda” (*n* = 1), “the LMIC is used merely as a data source” (*n* = 1), “they were only interested in data and not true capacity building” (*n* = 1), and “they had a condescending attitude and did not sufficiently value the input from researchers in the low-income country” (*n* = 1).

Both LMIC and HIC respondents noted that international collaboration provides access to something they otherwise would not have access to, including patient populations with diseases present only in LMICs and laboratory testing available in HICs. Respondents from both groups emphasized the essential and unique contributions of LMIC collaborators including understanding of local context and cultures, increased trust from study participants, enhanced study feasibility, and improved acceptability among study participants. Low- and middle-income country respondents observed that HIC collaborators contribute to improved study designs, organizational skills, data analysis, and clear scientific writing. Many LMIC respondents believed that involvement of HIC researchers enhanced the legitimacy and visibility of their work, making them more competitive for funding opportunities and enhancing the likelihood of publication in a higher impact journal. One LMIC respondent said:

One senses that journal editors are more likely to actually read the manuscript if HIC collaborators are involved.

A minority of the 252 respondents perceived that collaborative research with investigators from a country other than their own was harmful (8.7%, *n* = 22). Specifically, LMIC respondents mentioned bad publicity originating from the HIC collaborator, lack of respect from HIC collaborators, and not being included as an author despite meeting authorship criteria. High-income country respondents reported disadvantages associated with conducting research in a LMIC as such projects perceived to take longer to complete and may be at increased risk of non-completion because of potential unreliable collaborators in LMICs. One HIC respondent commented:

At times I have invested large amounts of time and financial resources in studies that were nearly complete, and then essential work by foreign collaborators was not done, in spite of dozens of reminders.

More than two-thirds of total respondents (67.5%, *n* = 170/252) perceived that HIC investigators had set the research agenda in previous work conducted in a LMIC, and this perception did not differ between respondents living in LMICs (*n* = 88, 65.2%) compared with those living in HICs (*n* = 82, 70.1%) (*p* = 0.407) (Table 2). The most commonly perceived reasons for HIC investigators setting the research agenda were access to funding (54.8%, *n* = 138), having had the idea for the study (48.0%, *n* = 121), and having published on the topic previously (32.1%, *n* = 81). Although the most commonly cited reason across all respondents that HIC investigators had set the research agenda was access to funding, when stratified by LMIC and HIC investigators, this perception did not differ significantly between LMIC (*n* = 69, 59.0%) and HIC (*n* = 69, 51.1%) investigators (*p* = 0.211). When compared with respondents from LMICs, respondents from HICs more commonly perceived that research agendas had been set by HIC investigators because they conceived the study idea (*p* < 0.001), had published on the topic previously (*p* = 0.011), had time to complete the study (*p* = 0.032), and had resources at their institutions to complete the work (*p* = 0.021). Among HIC investigators, the most commonly cited reason for HIC authors having set the research agenda was that they had the idea for the study (*n* = 72, 61.5%), whereas LMIC respondents most commonly felt that access to funding was the reason HIC investigators had set the research agenda (*n* = 69, 51.5%).

There were 131 unique, qualitative responses to the open-ended question soliciting opinions regarding steps to improve collaborative research efforts between LMIC and HIC investigators (76 from LMIC respondents and 55 from HIC respondents). Themes that emerged, corresponding frequencies of such responses, and representative quotes are found in Table 3. Main themes that emerged from the qualitative analysis were related to capacity building in research conducted in LMICs, collaboration principles that can guide successful collaborative work in LMICs, funding and its role in research agenda setting, appreciation for knowledge and skills among LMIC investigators, attitudinal changes among HIC investigators, and perception that collaborative work has improved over time.

**Table 3:** Summary of themes on improving collaborative research between investigators living in LMICs and HICs

Theme	Subthemes	LMIC respondents, *n* (*N* = 76)	HIC respondents, *n* (*N* = 55)	Direct quotes
Capacity building	Need for formal research training for LMIC investigators	8	12	“Improved training in research methodology in LMICs is likely to improve the quality of local research, foster greater collaboration, and promote equality in research recognition.” (LMIC respondent)
				“In the long term, increased training of junior LMIC researchers would have the highest payoff. In the short term, I’m not sure.” (HIC respondent)
	Experiential training	3	1	“Should be geared towards making researchers self-dependent after the research such that end products of research such as equipment etc. should be left behind. There should be lasting gains from the research that can empower such researchers.” (LMIC respondent)
				“The people who support data collection should be acknowledged and given opportunities to contribute to papers; however, they cannot claim to own intellectual property arising from the data.” (HIC respondent)
	Long-term collaboration	8	5	“Improving LMIC capacity to conduct research may not benefit directly a given study, but have important, long term consequences that should be in considered.” (LMIC respondent)
				“It has to be an explicit and sustained investment of time and resources in capacity-building for LMIC researchers.” (LMIC respondent)
				“Allowing for grace during the learning periods for both HIC and LMIC partners as both come to understand different portions of the research process.” (HIC respondent)
Collaboration principles	Mutual ownership from the onset	15	9	“The proposal must be ‘owned’ by all concerned from the inception. There should be equal ownership from inception of the project.” (LMIC respondent)
	Locally focused and sustainable	2	5	“Proactive steps by researchers from HICs to ensure the intellectual ‘center of gravity’ is in the countries affected by the problem being studied.” (LMIC respondent)
				“Research in LMIC should be conducted in collaboration with local stakeholders to ensure the success and durability/dissemination of the work. Without local stakeholder collaboration, a project is doomed to not be sustainable.” (HIC respondent)
	Equality in decision-making/lack of hierarchy	6	7	“A better appreciation that collaboration with researchers in LMICs must be built on mutual respect and interest in the science and that they are not just a vehicle for access to samples or participants.” (LMIC respondent)
				“All must be treated equally regardless of who holds the funding.” (HIC respondent)
	Established terms of reference	8	9	“Establishing from the beginning who is going to participate in the project and who is going to appear in the publication.” (LMIC respondent)
				“At a minimum, principles of collaboration should be set at the beginning of the project so that there is agreement on the roles and responsibilities.” (HIC respondent)
	Communication	3	8	“Having face to face work meetings to establish and maintain a working relationship.” (LMIC respondent)
				“The research should be known by all team members across the involved countries before starting…all of them should have the chance to modify objectives and methods, and to know their tasks and contributions in the project.” (HIC respondent)
Funding	Research funding	10	11	“Facilitating collaborative grant applications, with clear directive for use of funding in the study country.” (LMIC respondent)
				“One of the major problems in LMICs is the lack of funding for emerging researchers to remain researchers to develop their careers. High-income countries and universities could be particularly helpful in this regard.” (LMIC respondent)
	Funding for capacity building	0	2	“Funding for capacity building should be provided as a routine part of funded grants for LMICs to help those scientists improve. If funded projects just use those countries for recruiting, the scientists don't have the opportunity to gain all the type of experience a researcher needs.” (HIC respondent)
Appreciation for knowledge and skills among LMIC investigators	Respect for LMIC skills and knowledge	6	0	“Do not patronize, do not expect less from us. We want our science and our contribution to be as good as those from established research countries.” (LMIC respondent)
	Need for protected research time among LMIC investigators	4	4	“However, the immediate priority for LMIC physicians is often clinical care as there are too few providers for the population in need. In addition, they may not have the option of protected research time.” (LMIC respondent)
	LMIC investigators setting research agenda	10	5	“Researchers from high income countries should not conduct research based solely on their agenda and ideas but try to understand the local context in LMICs.” (LMIC respondent)
				“Encourage and fund researchers to formulate ideas based on experiences in low-income countries instead of only following the American/European research agenda for developing new products to be tested in low-income countries.” (LMIC respondent)
Attitude change	Avoid exploitation	5	3	“Authors from HICs feel securing that funding to conduct studies in LMICs also secures significant influence in running and micromanaging research units in LMICs.” (LMIC respondent)
				“I was not comfortable to see Clinical Research done under the U.S./European umbrella with LMICs execution roles only with a focus on cost saving by working there.” (HIC respondent)
	HIC investigator attitudes	5	3	“Researchers from developed countries should not think of themselves as ‘Gods’ of knowledge, but as collaborators in search for exchanging ideas. Native English speakers should have less prejudice to exchange ideas and studies with non-native English speakers—the last ones make (yes) grammar and spelling mistakes, but this does not diminish the quality of their work.” (LMIC respondent)
				“Mainly respect and less arrogance. All experts in the studied field have knowledge and a paternalistic and ‘imperialistic’ view is not the best way to dialogue. Sharing a research project must mean respect among the different views.” (HIC respondent)
	Avoid inferiority complex among LMIC investigators	3	0	“Researchers from LMICs would benefit from not feeling themselves always inferior, but as partners with good ideas.” (LMIC respondent)
Improvements over time		0	4	“Things have changed over the 30+ years I have worked in this field. In the early days, the idea and funding always came from a HIC, but LMIC-researchers were involved as PhD-students. Now it is usually more equal.” (HIC respondent)

HICs = high-income countries; LMICs = low- and middle-income countries.

## DISCUSSION

In our survey of pediatric investigators from across the world, most respondents perceived international collaborative research to be beneficial. However, more than two-thirds of respondents thought that HIC investigators had determined the agenda for research conducted in LMICs. Respondents identified important areas for improvement in academic global health collaborations including research capacity building among LMIC investigators, clear and frequent communication, and early involvement of LMIC investigators and shared decision-making during study conception and grant writing. In contrast to previous opinion pieces on academic global health collaborations, our study had a large number of LMIC respondents and provides one of the first pieces of evidence on LMIC respondent perceptions of academic global health collaboration.

Previous work suggests that advantages from academic global health collaborations include increased authorship opportunities, increased access to grant funding by LMIC investigators, and facilitation of academic promotion. However, these benefits accrue disproportionately to HIC investigators.^16,34,35^ Nevertheless, in our study, nearly 90% of both LMIC and HIC respondents perceived academic global health collaboration to be beneficial as a whole. However, LMIC respondents were less likely to report collaborative pediatric research as beneficial than HIC investigators, perhaps reflecting the maldistribution of benefits in collaborative research. Respondents indicated bidirectional knowledge exchange, varied perspectives, access to certain resources, long-term personal connections between collaborators, increased publications, and opportunities for career advancement as benefits derived from academic global health collaborations. Low- and middle-income country researchers in some cases perceived that collaboration with a HIC researcher was necessary to increase the visibility and legitimacy of their work. This may be due to underrepresentation of LMIC editorial board members and perceived biases in medical journals against work conducted in LMICs.^36–39^ In an opinion piece published in 2017, HIC authors advocated that bidirectional exchanges of personnel from LMICs and HICs provide meaningful benefits by strengthening ethical partnerships, furthering the education of the clinical workforce, and empowering trainees.^40^ Another study suggested that benefits of academic global health partnerships include publication of research, innovation and problem-solving from different perspectives, alliances promoting social justice, and enhanced capacity to scale-up effective interventions.^41^

Although respondents in our study believed such partnerships to be beneficial overall, some respondents reported challenges in academic global health collaborations. Specifically, LMIC respondents emphasized that HIC collaborators should not patronize them and that some HIC investigators needed overall attitudinal changes in collaborative work with a shift away from arrogance toward increased respect. Such sentiments echo the oftentimes lopsided nature of collaborative work that has been described in opinion pieces previously.^12,14,16^ Furthermore, a large proportion of respondents reported that HIC investigators had set the agenda for research conducted in LMICs. This finding aligns with comments published 20 years ago in the *BMJ* and echoed again in a more recent policy piece on inclusivity in global health published in 2017.^42,43^ Others have stressed the importance of locally led research in LMICs.^44^ The unintended consequences of HIC investigators setting the research agenda for collaborative work in LMICs are myriad. Without in-depth understanding of long-term availability of diagnostics and therapeutics, research can be misaligned with the implementation of new findings from research conducted in LMICs. Furthermore, conforming to external research agendas can disempower investigators in LMICs. Understanding local context, politics, and implications of research is paramount to conducting meaningful and ethical research in LMICs.

HIC investigator access to funding was the most commonly cited reason that HIC investigators set the research agenda. This may be related to challenges in accessing grant funding among LMIC investigators from complex grant applications, language barriers, and the fact that many LMICs do not have national funding agencies like the NIH in the United States.^2^ Although funding agencies such as the Wellcome Trust and the U.S. NIH have created opportunities for LMIC investigators to further build research capacity, recently, these initiatives have undergone funding cuts.^45–47^ Advocacy for the creation of, and support for, national funding agencies in LMICs could reduce the reliance on external funding agencies based in HICs.

Some HIC respondents perceived LMIC investigators’ lack of follow-through on essential research tasks as a barrier to completing studies. However, this perception fails to consider the factors that contribute to these barriers such as busy clinical and administrative load of many LMIC investigators (as denoted by our qualitative responses), sometimes limited Internet access, and lack of protected research time among LMIC investigators.^48,49^ Lack of protected time for research among LMIC investigators may be the result of a paucity of funding opportunities in LMICs. Increased funding for salary support to LMIC investigators may help overcome this barrier. Programs such as the NIH Fogarty International Center Global Health Training program fund both LMIC and HIC investigators to give them protected time for mentored research conducted in LMICs.^50^ Graduates of such programs gain requisite skills to conduct meaningful research that influences national and global policies and go on to receive further funding.^51,52^ Mutual understanding of local context may allow for more sustainable and understanding academic global health collaborations.

Our results add to the call for HIC investigators to encourage equal footing for LMIC investigators engaging in academic global health collaborations. Similar to a recently published qualitative study of 22 HIC and LMIC researchers,^53^ a report from a group at the Centre for Global Health at Trinity College in Ireland,^54^ and the 2014 ESSENCE on Health Research report from UNICEF, UNDP, the World Bank Group, and the WHO,^55^ themes from qualitative responses in our study included the need for capacity building, communication, and the importance of early involvement of both LMIC and HIC investigators along with shared decision-making at the time of study conception and grant writing. Studies comparing the output and long-term sustainability of academic global health collaborations built on evidence-based frameworks to those without such frameworks are needed. The recently launched Sustaining Technical and Analytic Resources initiative is a 5-year project supported by the United States Agency for International Development (USAID) to further determine “what works and what does not in creating and sustaining mutually benefitting academic partnerships” in LMICs.^56^ This type of practical work may serve as a model for mutually beneficial global health collaborations.

### Limitations.

The results of this study should be understood in the context of its limitations. Although we sent the survey to respondents more than once, provided incentive for respondents, and minimized the number of questions, all in-line with standard methods to increase response rates,^29,30^ we had a relatively low survey response rate with less than 20% of potential respondents completing the survey. The low response rate may have been due to the survey being online, surveying authors over a 10-year period during which investigators may have changed email addresses, and investigators dying or leaving academic appointments. Nevertheless, respondents and non-respondents did not differ by affiliated country income group, degree, or authorship position, and our response rate aligns with similar online survey studies.^32^ There is also the possibility that there was selection bias among respondents, and we were unable to determine if respondents’ opinions were significantly different from those who did not respond to the survey. Classifying respondents based on their current country could potentially misclassify some HIC respondents as respondents from LMICs if they expatriated to a LMIC, and vice versa. However, three-fourths of respondents had the same current country and birth country. Moreover, we had a higher proportion of responses from investigators from HICs among potential respondents, perhaps reflecting increased willingness to opine on academic global health collaborations. However, there was similar representation of LMIC and HIC investigators among respondents. Furthermore, our study was not powered to make comparisons for respondents from low-income countries alone, so we limited our comparison to investigators in LMICs grouped together. Further studies are merited to describe the perceptions of investigators in low-income countries as these may differ from those of investigators in middle-income countries.

Although we asked specifically whether HIC investigators had set the research agenda in work conducted in LMICs, our quantitative responses regarding benefits and harms from international collaborations did not differentiate HIC–LMIC collaborations from LMIC–LMIC collaborations. However, qualitative responses were indeed focused on HIC–LMIC collaboration. Moreover, responses to our survey may reflect recent experiences and opinions even among those who expatriated as they likely responded based on their environment at the time of the survey. Furthermore, as respondents had published articles as early as 2006, it is possible that there was recall bias among investigators who may not continue to be actively involved in collaborative academic global health work.

## CONCLUSION

Among pediatric investigators, academic global health collaborations were widely perceived as positive. However, most respondents felt that HIC investigators had set the research agenda for work conducted in LMICs. Respondents made suggestions for improving pediatric academic global health collaborations including research capacity building among LMIC investigators, frequent and respectful communication between HIC and LMIC investigators during studies, and the importance of early involvement of both LMIC and HIC investigators along with shared decision-making at the time of study conception and grant proposal writing.

## Supplemental table and appendix

Supplemental materials
