# Supplementary material for: Improving Pediatric Academic Global Health Collaborative Research and Agenda Setting: A Mixed-Methods Study
Source: Am J Trop Med Hyg. 2020 Jan 13;102(3):649–57. doi: 10.4269/ajtmh.19-0555 (PMC7056414; doi:10.4269/ajtmh.19-0555)
Supplement: Supplementary file 1 [file tpmd190555.SD1.pdf]

Supplemental Table 1. Comparison of responding authors to non-responding authors

	**Responding Authors, n = 252**	**Non-Responding Authors, n = 1,168**	***P*-value***
		n (%)	
Corresponding author in article reviewed, n (%)	143 (56.7)	682 (58.3)	0.631
Authorship position in article reviewed, mean (±SD)	3 (±3.3)	3.8 (±3.5)	0.167
Degree(s) listed in article reviewed, n (%)			0.884
Medical doctorate	162 (44.6)	721 (46.3)	
Doctorate degree	96 (26.5)	432 (27.8)	
Bachelor of Medicine and Bachelor of Surgery	25 (6.9)	95 (6.1)	
Master's degree	56 (15.4)	223 (14.3)	
Bachelor's degree	3 (0.8)	14 (0.9)	
Other†	21 (5.8)	72 (4.6)	
Total	363	1,557	
Listed affiliation with institution in: n (%)			0.018
High-income country	111 (44.0)	400 (34.2)	
Upper-middle income country	97 (38.5)	505 (43.2)	
Lower-middle income country	31 (12.3)	204 (17.5)	
Low-income country	13 (5.2)	59 (5.1)	

*Test statistic is Pearson chi-square test for all proportions. Student t-test used to compare mean authorship position in articles reviewed.

†Other highest degrees included fellowship (n=13 for respondents, n=40 for non-respondents), RN (n=0 for respondents, n=5 for non-respondents), DNB (n=1 for respondents, n=4 for non-respondents), DDS (n=0 for respondents, n=3 for non-respondents), DTMH (n=2 for respondents, n=3 for non-respondents), DVM (n=2 for respondents, n=3 for non-respondents), BDS (n=0 for respondents, n=2 for non-respondents), PharmD (n=1 for respondents, n=4 for non-respondents), RD (n=0 for respondents, n=2 for non-respondents), MHPE (n=1 for respondents, n=0 for non-respondents), MFPHM (n=0 for respondents, n=1 for non-respondents), PA (n=0 for respondents, n=1 for non-respondents), physical therapist (n=0 for respondents, n=1 for non-respondents), ND (n=0 for respondents, n=1 for non-respondents), MM (n=0 for respondents, n=1 for non-respondents), and DABP (n=0 for respondents, n=1 for non-respondents).
