# Supplementary material for: Improving Pediatric Academic Global Health Collaborative Research and Agenda Setting: A Mixed-Methods Study
Source: Am J Trop Med Hyg. 2020 Jan 13;102(3):649–57. doi: 10.4269/ajtmh.19-0555 (PMC7056414; doi:10.4269/ajtmh.19-0555)
Supplement: Supplementary file 2 [file tpmd190555.SD2.pdf]

Supplemental Appendix 1. Survey on collaboration in academic global health

DEMOGRAPHICS

In which country were you born?

a. Dropdown menu of all countries

In which country do you live currently?

a. Dropdown menu of all countries

What is your gender?

a. Male
b. Female
c. Other

What is your age in completed years? ________________________

Is English your primary language?

a. Yes
b. No

For respondents whose primary language is not English: What is your primary language?

What is your highest degree? Choose one.

a. Medical doctorate (MD)
b. Doctorate degree (e.g. doctorate of philosophy: PhD, doctorate of science: ScD, doctorate of public health: DrPH, or other doctorate degree)
c. Medical doctorate (MD) and doctorate degree (e.g. PhD, ScD, DrPH, etc.)
d. Bachelor of medicine and bachelor of surgery (MBChB, MBBS)
e. Bachelor of medicine and bachelor of surgery (MBChB, MBBS) and doctorate degree (e.g. PhD, ScD, DrPH, etc.)
f. Master's degree (e.g. MPH, MHS, MSc, etc.)
g. Bachelor's degree (not medical)
h. Other

In what country did you do your medical training?

a. Dropdown menu of all countries

In what country did you complete your highest degree?

a. Dropdown menu of all countries

Which of the following best describes your current position? Select all that apply.

a. Student
b. Medical resident
c. Fellow (i.e. a person who has finished residency and is pursuing sub-specialty training)

d. Clinical instructor
e. Assistant professor
f. Associate professor
g. Full professor
h. Lecturer
i. Reader
j. Medical doctor with no academic appointment
k. Clinical officer
l. Medical officer
m. Employed by a pharmaceutical company
n. Employed by a non-governmental organization
o. Support staff
p. Other. Please describe your current position. ____________________

How many peer-reviewed articles have you published in total? ____________________

INTERNATIONAL COLLABORATION

Have you ever collaborated with a researcher, or researchers, who is/are from a country other than your own?

a. Yes
b. No

Has collaborating with researchers from countries other than your own benefitted you?

c. Yes
d. No
e. I don't know

For respondents who reported benefits from collaborating with researchers from countries other than their own:

How has collaborating with researchers from countries other than your own benefitted you?

____

Has collaborating with researchers from countries other than your own harmed you?

a. Yes
b. No
c. I don't know

For respondents who reported harm from collaborating with researchers from countries other than their own:

How has collaborating with researchers from countries other than your own harmed you?

____

On any of your previous collaborative work, have you felt that an author from a high-income country defined the research agenda?

a. Yes
b. No
c. I don't know
d. I have never worked with a researcher from a high-income country

For respondents who reported high-income collaborators defined the research agenda:

Why do you think the researcher from a high-income country defined the research agenda? Select all that apply.

a. They had access to funding for the study
b. They had the idea for the study
c. They had published on that topic previously
d. They had the time to complete the study
e. They had resources at their institution to complete the study
f. Other
    i. Please describe why the researcher from a high-income country defined the research agenda.

    ____

Please provide your opinion regarding steps that can be taken to improve collaborative research efforts between authors from high-income countries and low- and middle-income countries.

____
